# Global research status and trends in macrodactyly research: Bibliometric and visualized analysis from 2005 to 2025

**DOI:** 10.1097/MD.0000000000048130

**Published:** 2026-03-20

**Authors:** Yuan Liu, Zong-You Yang, Chao-Jian Pang, Xiao-Bo Fan, Chen-Yang Zhao, Zhi-Kun Wei

**Affiliations:** a Department of Orthopaedic Surgery, The First Hospital of Handan, Handan, Hebei Province, China; b Department of Orthopaedic Surgery, The Third Hospital of Hebei Medical University, Shijiazhuang, Hebei Province, China.

**Keywords:** hotspot, macrodactyly, management, overgrowth, visualized

## Abstract

**Background:**

To provide a comprehensive bibliometric and visualized analysis of global macrodactyly research from 2005 to 2025, identify publication trends, leading contributors, research hotspots, and emerging directions in this rare congenital disorder.

**Methods:**

Publications from January 1,2005 to November 31,2025 were retrieved from the Web of Science Core Collection. English-language articles and reviews were included using the search terms “macrodactyly,” “megalodactyly,” “digital gigantism,” and “giant digit.” After removing duplicates, retracted items, and non-relevant records, eligible studies were analyzed. Microsoft Excel, VOSviewer, and CiteSpace were used to evaluate publication trends, geographic distribution, collaboration networks, and keyword co-occurrence patterns.

**Results:**

A total of 162 publications met the inclusion criteria. Annual output increased steadily, with peaks in 2014 and 2020, and a strong upward cumulative trend (*R*^2^ = 0.9933). Research was mainly concentrated in the USA, China, and Europe, with limited intercontinental collaboration. Author and institutional analyses revealed several distinct collaboration clusters. Keyword co-occurrence and temporal mapping demonstrated a shift from early clinical and surgical topics toward molecular and genetic research, particularly involving PIK3CA-related mechanisms.

**Conclusion:**

Macrodactyly research has expanded over the past 2 decades, evolving from clinical descriptions to mechanistic studies driven by advances in molecular genetics. The identification of PIK3CA mutations has reshaped the field and introduced opportunities for targeted therapy. Despite increasing output, international collaboration remains limited. Future work should focus on multicenter studies, precision medicine approaches, and the development of evidence-based treatment strategies.

## 1. Introduction

Macrodactyly is a rare congenital condition characterized by the overgrowth of digits involving both soft tissue and osseous structures.^[1,2]^ This condition typically presents at birth and progresses throughout childhood, causing significant functional impairment. The incidence of macrodactyly is estimated to be approximately 1 in 100,000 live births, representing approximately 0.9% of congenital anomalies affecting the upper extremities.^[3]^ The etiology of macrodactyly remained poorly understood for decades until recent advances in molecular genetics revealed its underlying mechanisms. Phosphatidylinositol-4,5-bisphosphate 3-kinase catalytic subunit alpha (PIK3CA) gene encodes a protein that plays a crucial role in the phosphoinositide 3-kinase/protein kinase B signaling pathway, which controls cell growth, proliferation, and survival.^[4,5]^ Recent molecular genetics studies have identified PIK3CA mutations as a central mechanism in macrodactyly pathogenesis.^[6]^ Clinically, macrodactyly presents considerable challenges in both diagnosis and management. The condition is typically classified into 2 types: static macrodactyly, where the enlarged digit grows proportionally with the rest of the body, and progressive macrodactyly, where the affected digit grows disproportionately faster.^[7]^ Treatment remains primarily surgical, including debulking procedures and amputation. However, the emergence of targeted therapies such as PIK3CA inhibitors has opened new avenues for non-surgical management.

Bibliometric and visualized analysis has become an increasingly valuable tool for evaluating scientific research output and identifying trends within specific fields.^[8]^ By systematically analyzing publication patterns, author collaborations, and keyword co-occurrences, bibliometric studies provide insights into the evolution and current state of research in a field. This methodology enables researchers to identify influential works, emerging hotspots, and future research directions. Understanding publication trends, identifying leading contributors, and recognizing emerging research themes are essential for guiding future investigations and clinical practice. In addition, visualized analysis enables researchers to identify potential collaborators and promote international cooperation.

This study aims to conduct a systematic bibliometric and visualized analysis of macrodactyly research published between 2005 and 2025, using visualization tools to map the scientific landscape, identify research hotspots, and explore potential future directions in this field.

## 2. Methods

### 2.1. Search strategy and data collection

All publications included in this study were sourced from the Web of Science Core Collection (WoSCC). The literature search was conducted on December 1, 2025. We restricted our analysis to English-language publications to minimize potential translation bias. The selection criteria were: English-language publications; published from January 1, 2005 to November 30, 2025; document types restricted to articles and review; and search terms: “macrodactyly,” “megalodactyly,” “digital gigantism,” and “giant digit.” All retrieved records were downloaded in “plain text” format to capture relative information, such as titles, abstracts, author names, keywords, publication years, journal sources, and countries or regions. Records with incomplete data were excluded. Duplicate and retracted items were identified through automated cross-checking of titles and authors and were subsequently removed. A secondary screening phase was performed manually by reading the titles, authors, and abstracts of each record. Publications categorized as conference abstracts, other forms of gray literature, letters, editorial notes, corrections, book chapters, or those unrelated to macrodactyly were further assessed and excluded. The literature search and data extraction were independently completed by 2 reviewers, and a third reviewer assessed the final dataset for consistency. All data were retrieved from publicly available sources and did not involve clinical information or human participants. The institutional ethics approval was not required.

### 2.2. Statistical analysis and visualization

The software tools used in this study included Microsoft Excel 2021 (Microsoft Corp., Redmond), VOSviewer (version 1.6.20; Centre for Science and Technology Studies, Leiden University, Leiden, Netherlands), and CiteSpace (version 6.2.R4; Drexel University, Philadelphia). The functions available in Excel were used to organize the retrieved data and to generate graphical outputs, such as the annual publication trends and distribution of studies. The geographic distribution of publications was visualized using a world map, with color intensity reflecting the publication frequency of each country. Darker shades indicated higher research productivity across countries and regions. Linear regression analysis was performed to evaluate the growth trend of cumulative publications over time, with the coefficient of determination (*R*^2^) calculated to assess model fit.

CiteSpace was employed to construct network visualizations of international collaboration among countries, authors, and institutions. In these network maps, the nodes represented independent units. The size of each node represented the frequency and the links between nodes indicated collaborative relationships. For author and institutional collaboration analysis, a minimum threshold of 2 publications was applied. VOSviewer was used to construct visualizations of keyword co-occurrence. In these maps, nodes represented keywords, and the size of each node represented the frequency of occurrences, whereas the links between nodes indicated relationships. Different colors in the network visualization represented distinct clusters. For keyword co-occurrence analysis, a minimum threshold of 5 occurrences was set. The overlay visualization function in VOSviewer was employed to analyze the evolution of research themes. In overlay maps, colors represented the publication year of each keyword, with purple-blue indicating earlier research focus and green-yellow representing more recent research topics.

## 3. Results

**Figure 1.**
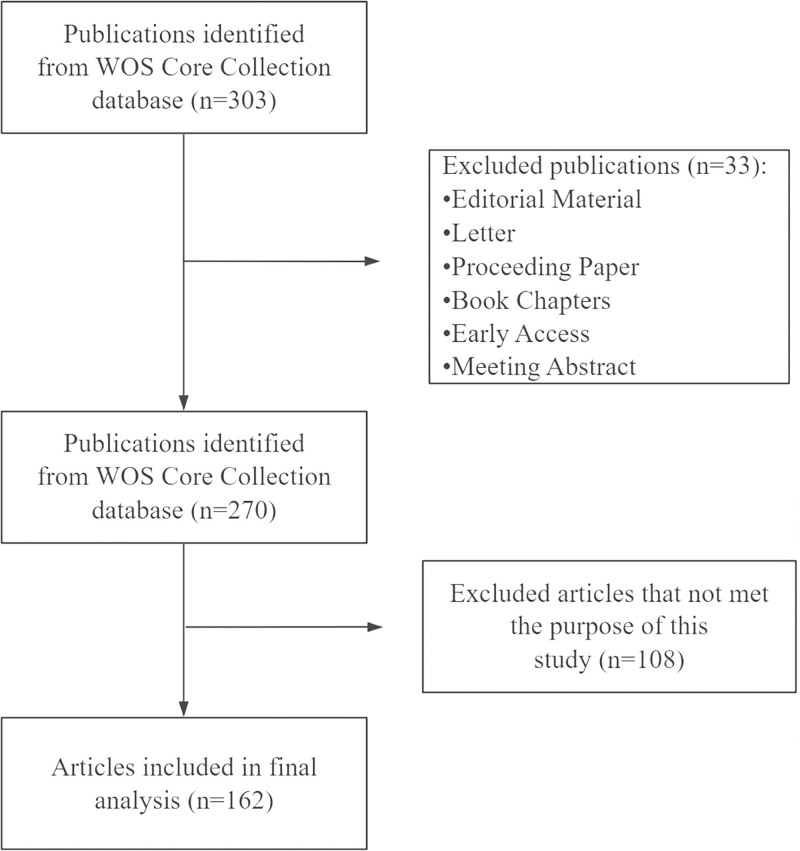
Flowchart of the literature screening process. A total of 303 publications were initially identified. The selection criteria were: (1) English-language publications; (2) published from January 1, 2005 to November 30, 2025; (3) document types restricted to articles and review; and (4) search terms: “macrodactyly,” “megalodactyly,” “digital gigantism,” and “giant digit.” Final analysis included 162 articles.

**Figure 2.**
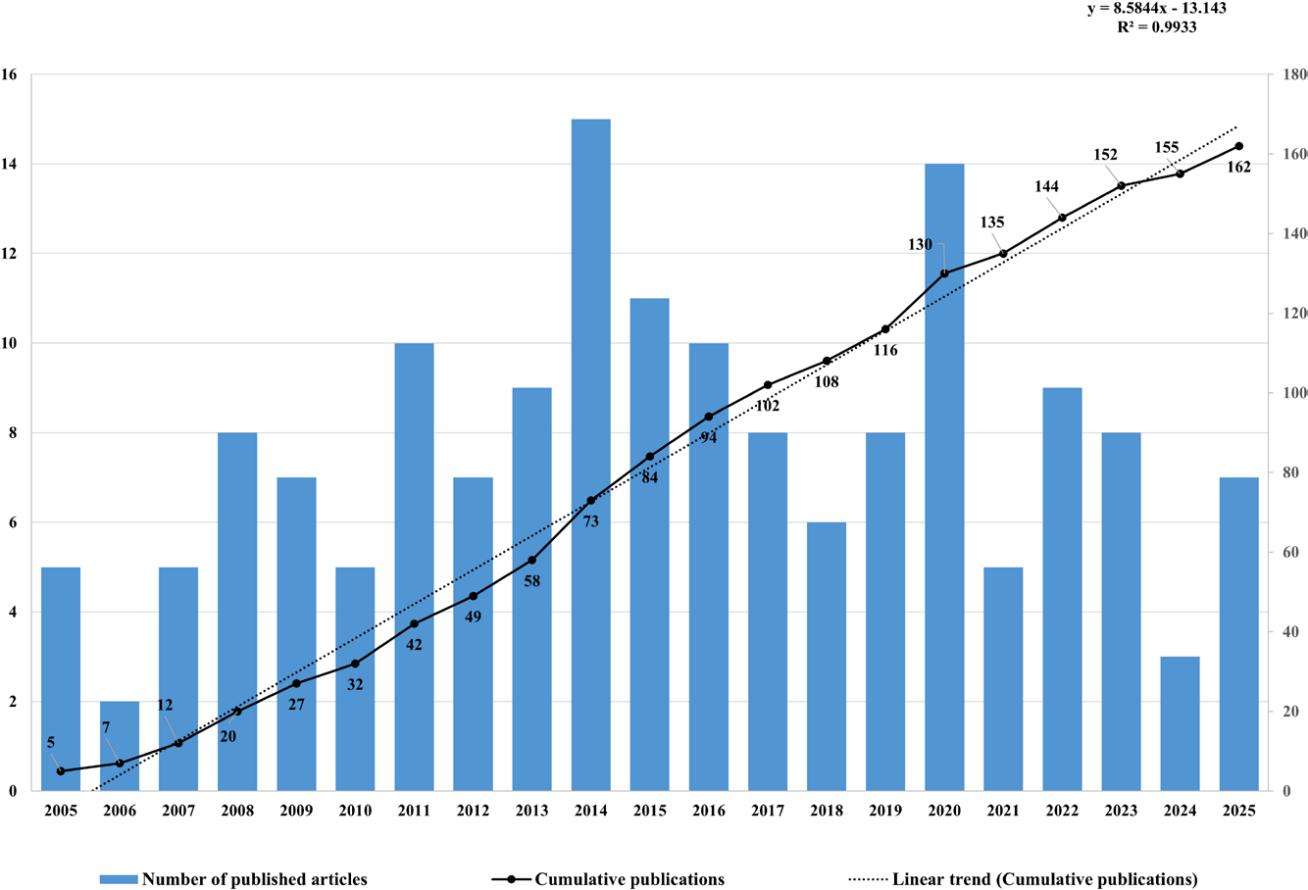
Annual publication trends of macrodactyly research from 2005 to 2025. The blue bars represent the number of published articles per year, while the solid line with dots indicates the cumulative number of publications. The dotted line represents the linear trend of cumulative publications. Linear regression analysis revealed a strong positive correlation (*y* = 8.5844*x* − 13.143, *R*^2^ = 0.9933).

We screened and included 303 publications where initially retrieved from the WoSCC database. After excluding 33 non-article documents, 270 publications remained. Following manual screening to exclude 108 irrelevant articles, 162 articles were included in the final analysis (Fig. 1). The annual publication trend from 2005 to 2025 was shown in Fig. 2. Annual output ranged from 2 to 15 articles, with peak years in 2014 (*n* = 15) and 2020 (*n* = 14). The cumulative publication curve demonstrated steady growth, increasing from 5 in 2005 to 162 by 2025. Linear regression analysis revealed a strong positive trend (*R*^2^ = 0.9933, *y* = 8.5844*x* − 13.143), indicating consistent growth in macrodactyly research over the past 2 decades.

### 3.1. Analysis of national publications and collaboration

**Figure 3.**
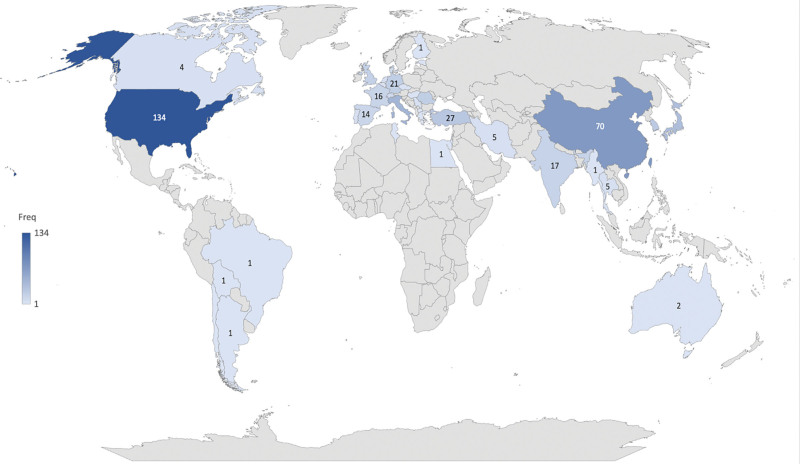
Geographic distribution of macrodactyly publications worldwide. The color intensity reflects the publication frequency of each country, with darker shades indicating higher research productivity.

The geographic distribution of macrodactyly research was illustrated in Fig. 3. A total of 41 countries contributed to the 162 publications. The USA ranked first with 134 publications, followed by China and Italy as major contributors. Other countries including Canada and Australia also participated in macrodactyly research with fewer publications. The research output was mainly concentrated in North America, Asia, and Europe. These contributions from Africa and South America remained limited.

**Figure 4.**
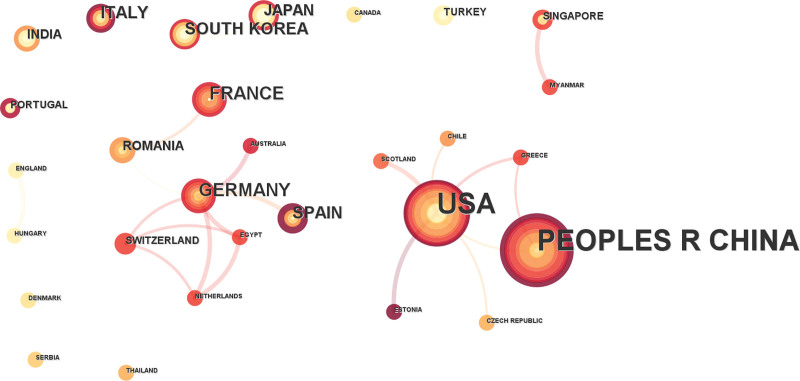
Network visualization of international collaboration among countries in macrodactyly research. Each node represents a country, with node size proportional to publication output. The links between nodes indicate collaborative relationships between countries. The network reveals 2 major collaboration clusters centered around the USA–China and European countries.

The international collaboration network was presented in Fig. 4. The USA and China emerged as the 2 major collaboration centers. The USA established collaborative links with multiple countries including China, Greece, and Scotland. European countries formed another collaboration cluster. Among them, Germany was the center of the research, connecting Spain, Switzerland, the Netherlands, and Romania. Asian countries, including India, Thailand, Japan, South Korea, and Singapore, showed less active collaboration.

### 3.2. Collaboration analysis of authors

**Figure 5.**
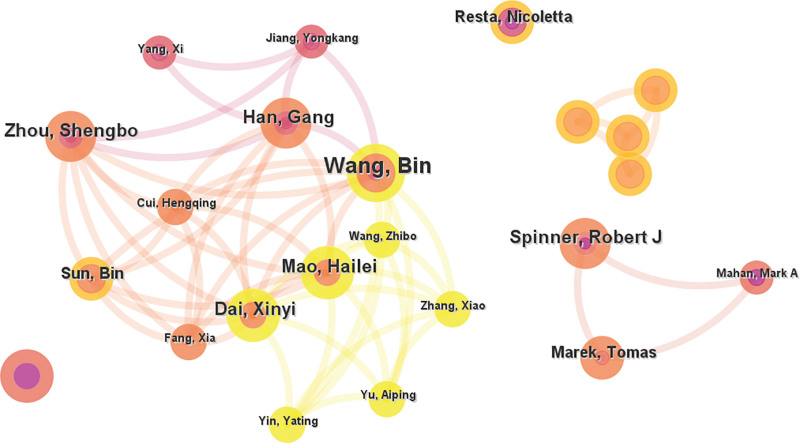
Collaboration network of authors in macrodactyly research. Authors with at least 2 publications were included. Each node represents an author. Different colors represent distinct collaboration clusters.

The author collaboration network was presented in Fig. 5. Among the 762 authors involved in macrodactyly research. Instead of analyzing collaboration among all authors, we focused on those with more than 2 publications, as they were more experienced in this field. A total of 50 authors met the threshold of at least 2 publications. The network revealed several distinct collaboration clusters. The largest cluster was centered around Wang Bin, who collaborated closely with Mao Hailei, Dai Xinyi, Han Gang, Zhou Shengbo, and Sun Bin. They were forming a connected Chinese research team. Another notable cluster included Spinner Robert J. and Marek Tomas with their collaborators. However, Resta Nicoletta demonstrated fewer collaborations, as he appeared among the authors with only one publication.

### 3.3. Analysis of institutional collaborations

**Figure 6.**
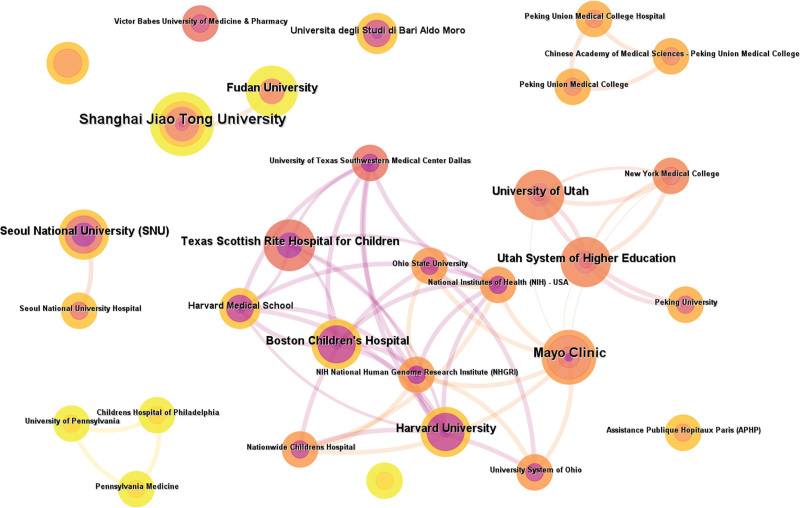
Institutional collaboration network in macrodactyly research. Institutions with at least 2 publications were included. Each node represents an institution.

The institutional collaboration network of macrodactyly research was presented in Fig. 6, including institutions with more than 1 publications. The network revealed several major collaboration clusters. American institutions formed the most densely connected cluster, with Boston Children Hospital, Harvard University, Mayo Clinic, and Texas Scottish Rite Hospital for Children serving as central parts. These institutions established extensive collaborative links with National Institutes of Health, University of Utah, and Ohio State University. Chinese institutions formed another prominent team, led by Shanghai Jiao Tong University and Fudan University, along with Peking Union Medical College and affiliated hospitals. Seoul National University represented active collaboration in Asia. European institutions, including Victor Babes University, formed a smaller cluster.

### 3.4. Analysis of keyword co-occurrence

**Figure 7.**
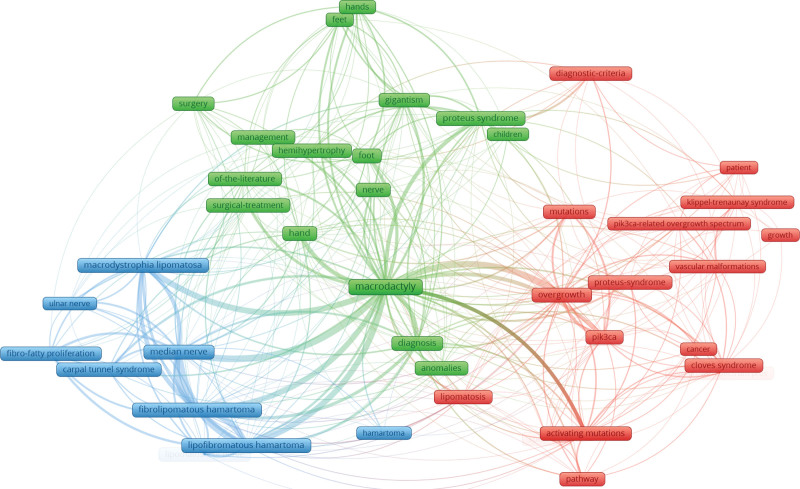
Keyword co-occurrence network visualization in macrodactyly research. Keywords with at least 5 occurrences were included. Each node represents a keyword, with node size reflecting occurrence frequency. The links between nodes indicate co-occurrence relationships. The colors represent 3 distinct clusters.

The network visualization of keyword co-occurrence was presented in Fig. 7. Among 641 keywords identified, 42 met the threshold of at least 5 occurrences. The network revealed 3 distinct clusters. The green cluster focused on clinical features and treatment. This cluster primarily reflects clinical diagnosis and surgical management of macrodactyly. The blue cluster focused on pathological features and associated conditions. The red cluster emphasized genetic and molecular mechanisms, featuring keywords such as PIK3CA, activating mutations, and overgrowth. This cluster indicated growing research on the genetics of macrodactyly.

**Figure 8.**
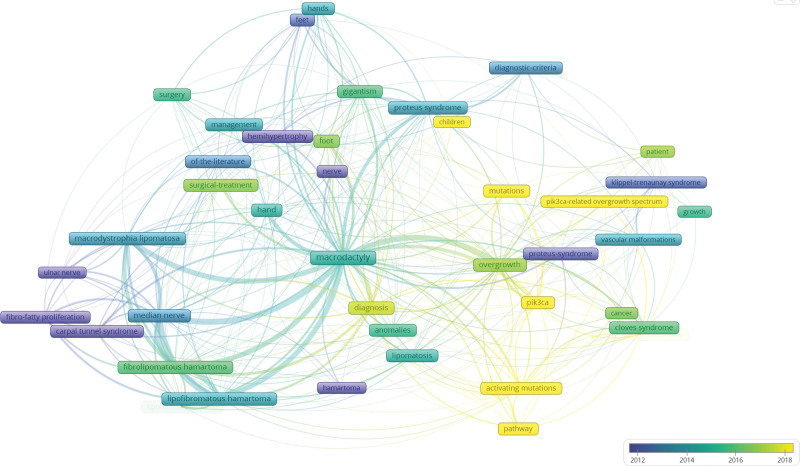
Overlay visualization of keyword co-occurrence network. The color scale indicates the publication year of each keyword, ranging from purple to yellow (2005–2025).

The overlay visualization of keyword co-occurrence (Fig. 8) demonstrated the evolution of research focus. Earlier research (purple-blue, before 2014) concentrated on clinical descriptions such as surgery and diagnostic criteria. The intermediate period (blue-green, 2014–2016) showed increasing attention to pathological features including nerve involvement. More recent studies (green-yellow, 2016–2018) shifted their focus toward mechanisms of overgrowth. The most recent keywords (yellow, after 2018), such as mutations, pathway, and PIK3CA-related overgrowth spectrum, represent current research frontiers. This trend was further supported by the latest publications in 2024 to 2025, which have explored the mechanisms by which activating PIK3CA mutations promote adipose tissue overgrowth through inhibiting lipophagy, investigated how PIK3CA mutations in adipose-derived stem cells drive phenotypic changes.^[9–11]^

## 4. Discussion

This visualized and bibliometric analysis provides a comprehensive overview of macrodactyly research from 2005 to 2025, revealing significant growth in this field. The number of publications remained steady, reflecting the consistent scientific interest in this rare congenital disease. The peaks in publication numbers in 2014 and 2020 may be related to important progress in understanding the genetic basis of macrodactyly. In particular, the discovery of somatic PIK3CA mutations by Lindhurst et al^[5]^ provided important insights into the molecular mechanisms.

The steady increase in cumulative publications, with an annual growth rate of approximately 8.58 articles per year (*R*^2^ = 0.9933), indicates that research on macrodactyly has progressed toward more comprehensive investigation. This growth reflects an expanding research focus, including advances in diagnostic technologies and increasing attention to molecular and genetic mechanisms that provide deeper insights into the condition. However, the total number of publications-only 162 over 2 decades-highlights the inherent challenges of studying a rare disorder, such as limited patient availability and constraints in research funding. To address these barriers, patient registries play a critical role in rare-disease research.^[12,13]^ Establishing multicenter registries would allow for larger sample sizes, long-term follow-up, and enhanced data sharing among researchers worldwide. Therefore, greater efforts should be directed toward developing such registries to further advance research on rare diseases.

Our analysis revealed a marked geographic concentration of macrodactyly research, with the USA and China emerging as dominant contributors. This distribution pattern is consistent with the broader landscape of biomedical research, in which resource-rich countries with well-established research infrastructures. Such environments provide greater support for sustained scientific output. American institutions are highly represented in this field. Specialized pediatric centers such as Boston Children Hospital and Texas Scottish Rite Hospital for Children play a major role. This reflects their strong clinical experience and research ability. Similarly, Chinese institutions including Shanghai Jiao Tong University and Fudan University have emerged as significant contributors, consistent with China rapid expansion in medical research output over the past 2 decades.

The collaboration network demonstrated clear regional clusters, with limited cooperation across continents. European institutions formed a central group around Germany, while Asian countries maintained independent collaborative networks. This pattern indicates that, despite the global nature of scientific exchange, research on macrodactyly remains largely concentrated within regional settings. Strengthening international collaboration could accelerate research progress by facilitating patient recruitment for multicenter studies, sharing rare tissue samples, and pooling expertise across different specialties.^[14,15]^ As emphasized by Julkowska et al, collaborative efforts are particularly crucial for rare diseases where individual centers may encounter insufficient case numbers to draw meaningful conclusions.^[16]^

The keyword co-occurrence analysis revealed a clear thematic evolution in macrodactyly research over the period. Early research predominantly focused on clinical descriptions and surgical management. This pattern reflects the traditional approach to rare congenital conditions, where initial understanding typically derives from careful clinical observation. Terms such as “surgical treatment” and “management” appeared frequently in the early literature. This suggests that the primary focus during that period was on surgical approaches to treatment. The intermediate period witnessed increasing attention to pathological features. These conditions, characterized by excessive fibro-fatty proliferation along affected nerves, represent key histopathological hallmarks of macrodactyly. The association between macrodactyly and nerve-related complications became increasingly recognized during this period. The increasing appearance of keywords such as “PIK3CA,” “activating mutations,” and “PIK3CA-related overgrowth spectrum” demonstrates the field move toward molecular genetic research. The discovery of somatic mosaic PIK3CA mutations as the cause of macrodactyly was a major breakthrough. It classified macrodactyly as part of the wider PIK3CA-related overgrowth spectrum.^[17]^ This molecular insight has important implications for diagnosis and treatment. It allows genetic confirmation of clinical findings and provides new opportunities for developing targeted therapies. The phosphoinositide 3-kinase–protein kinase B–mTOR signaling pathway has emerged as the central molecular mechanism underlying macrodactyly pathogenesis.^[18]^ Activating mutations in PIK3CA leads to constitutive activation of this pathway, resulting in enhanced cell proliferation, survival, and metabolism.^[19]^ Recent studies have further elucidated the downstream consequences of PIK3CA activation in affected tissues.^[20]^ The latest research published in 2025 has shown that activating PIK3CA mutations promote adipose tissue overgrowth by inhibiting lipophagy, with USP15 identified as a potential therapeutic target.^[6]^

These mechanistic insights have significant therapeutic implications. The traditional approach to macrodactyly management has relied primarily on surgical interventions, including amputation in severe cases.^[21–23]^ While surgery remains the mainstay of treatment, outcomes are often suboptimal due to recurrence of overgrowth and functional limitations. The identification of PIK3CA as a molecular target has spurred interest in pharmacological approaches. Venot et al reported successful treatment of patients with PIK3CA-related overgrowth spectrum disorders using PIK3CA inhibitor, demonstrating reduction in tissue overgrowth and functional improvement.^[24]^ This landmark study established proof of concept for targeted therapy in PIK3CA-related conditions, including macrodactyly. The translation of molecular discoveries into clinical applications represents a promising frontier in macrodactyly management. However, several challenges remain, including the optimization of drug dosing in pediatric populations, long-term safety considerations, and identification of patients most likely to benefit from targeted therapy.

Based on our bibliometric analysis, several research hotspots and future directions can be identified. First, understanding the phenotypic spectrum associated with different PIK3CA mutations and their tissue-specific effects will be crucial for developing precision medicine approaches.^[25]^ Second, the role of the tumor microenvironment and cellular crosstalk in tissue overgrowth warrants further exploration. Recent studies have demonstrated that PIK3CA-mutant cells can influence the behavior of surrounding wild-type cells, suggesting that paracrine signaling mechanisms may contribute to disease progression.^[26]^ Third, the development of novel surgical techniques continues to advance. The recent description of toenail composite tissue flap reconstruction for foot macrodactyly represents an innovative approach that may improve functional and aesthetic outcomes.^[27]^ Integrating molecular and genetic findings with surgical decision-making may enable more personalized treatment strategies, such as selecting appropriate resection margins and determining the need for staged procedures.^[28–31]^ Fourth, the potential association between PIK3CA mutations and malignancy risk remains an important area of investigation.^[32]^ Long-term follow-up studies are needed to determine whether patients with macrodactyly face elevated cancer risk and whether surveillance protocols are warranted.

## 5. Limitations

Several limitations of this study should be acknowledged. First, our analysis was restricted to the WoSCC database, which may have excluded relevant publications indexed in other databases such as Scopus or PubMed. However, Web of Science is widely recognized for its rigorous indexing standards and comprehensive coverage of high-quality scientific literature. Second, the bibliometric methodology inherently favors English-language publications, potentially underrepresenting research from non-English speaking regions. Finally, citation analysis may be influenced by factors unrelated to scientific merit, including self-citation practices and publication timing.

## 6. Conclusion

This bibliometric analysis provides a comprehensive overview of macrodactyly research over the past 2 decades, revealing steady growth in publication output and significant evolution in research themes. The field has progressed from predominantly clinical and surgical descriptions toward molecular mechanistic investigations, with PIK3CA mutations now recognized as the primary genetic driver of this condition. The USA and China have emerged as leading contributors, though international collaboration remains limited. Future research should focus on developing targeted therapies, and establishing evidence-based management guidelines. Enhanced international collaboration and multicenter studies will be essential for advancing our understanding of this rare condition.

## Acknowledgments

The authors used Claude (Anthropic) to assist with English language polishing and improving readability during manuscript preparation. All content was critically reviewed, verified, and revised by the authors, who take full responsibility for the final work.

## Author contributions

**Conceptualization:** Yuan Liu.

**Data curation:** Xiao-Bo Fan, Chen-Yang Zhao, Zhi-Kun Wei.

**Formal analysis:** Zong-You Yang.

**Funding acquisition:** Zong-You Yang.

**Project administration:** Xiao-Bo Fan.

**Supervision:** Yuan Liu.

**Writing – original draft:** Yuan Liu, Zong-You Yang, Chao-Jian Pang.

**Writing – review & editing:** Yuan Liu, Zong-You Yang, Chao-Jian Pang, Xiao-Bo Fan, Chen-Yang Zhao, Zhi-Kun Wei.
